# Clinical Significance of Mesenchymal Circulating Tumor Cells in Patients With Oligometastatic Hormone-Sensitive Prostate Cancer Who Underwent Cytoreductive Radical Prostatectomy

**DOI:** 10.3389/fonc.2021.812549

**Published:** 2022-01-20

**Authors:** Guanjie Yang, Jun Xie, Shun Zhang, Wenyu Gu, Jing Yuan, Ruiliang Wang, Changcheng Guo, Lin Ye, Bo Peng, Xudong Yao, Bin Yang

**Affiliations:** ^1^ Department of Urology, Shanghai Tenth People’s Hospital, Tongji University School of Medicine, Shanghai, China; ^2^ Shanghai Clinical College, Anhui Medical University, Shanghai, China; ^3^ Department of Urology, Affiliated Drum Tower Hospital, Medical School of Nanjing University, Nanjing, China

**Keywords:** oligometastatic prostate cancer, circulating tumor cell, epithelial–mesenchymal transition, androgen deprivation therapy, prostate-specific antigen, radical prostatectomy, liquid biopsy, biomarker

## Abstract

**Purpose:**

Growing evidence shows that circulating tumor cells (CTCs) become more aggressive after the epithelial–mesenchymal transition (EMT), though the clinical significance of CTCs undergoing EMT in oligometastatic hormone-sensitive prostate cancer (omHSPC) patients has not yet been reported. Accordingly, the aim of this study was to detect the CTC level and investigate the clinical significance of mesenchymal CTCs in omHSPC patients who underwent cytoreductive radical prostatectomy (CRP).

**Materials and Methods:**

Blood samples were drawn from 54 omHSPC patients who underwent CRP. The CanPatrol CTC enrichment technique was applied to isolate and identify different phenotypes of CTCs, which were classified as epithelial (E-CTCs), mesenchymal (M-CTCs), or biphenotypic epithelial/mesenchymal (Bi-CTCs). Univariable and multivariable Cox regression analyses were employed to investigate potential prognostic factors for metastatic castration-resistant prostate cancer (mCRPC)-free survival and cancer-specific survival (CSS). The prognostic value of CTCs for CSS and mCRPC-free survival was assessed using time-dependent receiver operating characteristic (ROC) curves and Kaplan–Meier analysis.

**Results:**

CTCs were detected in 51 of 54 patients (94%). E-CTC, M-CTC, and Bi-CTC detection rates were 56%, 67%, and 85%, respectively. A positive correlation was found between the M-CTC count and number of bone metastases (*p* = 0.012). Time-dependent ROC analysis showed that the M-CTC count had higher predictive power than E-CTC or Bi-CTC for mCRPC-free survival (3-year area under the curve [AUC] values: 0.64, 0.60, and 0.61) and CSS (3-year AUC: 0.86, 0.58, and 0.67). Additionally, time-dependent ROC analysis revealed total CTCs (T-CTCs) ≥5 and M-CTCs ≥2 to be the cutoff points with optimal specificity and sensitivity. Based on multivariable Cox regression, T-CTC and M-CTC counts were both independently associated with CSS and mCRPC-free survival (all *p* < 0.05), though E-CTCs and Bi-CTCs had no significant prognostic value (all *p* > 0.05). Patients with T-CTC ≥5 or M-CTC ≥2 had significantly worse mCRPC-free survival and CSS than those with T-CTC<5 or M-CTC<2 (all *p* < 0.05) after CRP.

**Conclusion:**

CTC quantification and phenotype characterization provide prognostic information, and M-CTCs can be used as a novel biomarker for omHSPC patients who undergo CRP. The results need to be validated in prospective studies.

## Introduction

The oligometastatic state has been recognized as an intermediate state between localized disease and widespread metastases, suggesting the potential for preventing additional metastatic spread and improving survival with local treatment (such as surgery and radiotherapy) (1, 2). Retrospective studies have reported that cytoreductive radical prostatectomy (CRP) reduces the risk of clinical progression and improves cancer-specific survival (CSS) (3–5). According to a recent prospective registry, CRP is able to improve CSS and overall survival (OS) in newly diagnosed low-volume metastatic prostate cancer patients (6). Despite improvements in diagnosis and treatment, omHSPC patients comprise a heterogeneous population of men with different outcomes who will ultimately develop castration-resistant cancer (7, 8). Thus, it is necessary to develop a novel biomarker that can better predict the prognosis of these patients.

Circulating tumor cells (CTCs), which are shed from solid tumors, are presumed to constitute the mechanism for cancer metastasis (9). To date, CellSearch is the only method that has been analytically validated and cleared by the Food and Drug Administration (FDA) for use. The CTC level has been regarded as a surrogate biomarker for survival in patients with mCRPC (10, 11). It was also reported that CTCs might contribute to identifying high-risk prostate cancer patients with occult metastases at the time of diagnosis (12). In addition, CTCs might provide prognostic information for omHSPC and help in the selection of patients for CRP (13). However, the CTC isolation and capture techniques mentioned above depend on epithelial markers (such as epithelial cell adhesion molecule, EpCAM) for E-CTCs, which may fail to detect M-CTCs (14, 15).

Growing evidence shows that CTCs become more aggressive after adopting a mesenchymal phenotype during the epithelial–mesenchymal transition (EMT) (16, 17). Although mesenchymal CTCs (M-CTCs) are associated with tumor progression and poor prognosis in many carcinomas (18, 19), to the best of our knowledge, detection of M-CTCs in omHSPC has not been documented, and it remains unclear whether M-CTCs are involved in the progression of omHSPC after CRP. In this study, we used the CanPatrol CTC enrichment technique, which has been applied for a broad range of carcinomas based on epithelial and mesenchymal markers (20–24), to detect the CTC level and to investigate the clinical significance of CTCs undergoing EMT in omHSPC patients treated with CRP.

## Materials and Methods

### Patient Population

A total of 54 patients with omHSPC who underwent CRP at Shanghai Tenth People’s Hospital of Tongji University from January 2015 to November 2017 were retrospectively enrolled in this study. All patients were examined by routine laboratory tests, serum prostate-specific antigen (PSA) and testosterone level measurement, thoracic, abdominal, and pelvic computed tomography (CT) scans, magnetic resonance imaging, and whole-body bone scan. The inclusion criteria were as follows: (a) resectable primary prostate cancer; (b) five or fewer bone metastases with or without suspicious pelvic nodal involvement confirmed by bone scan, CT scan, or magnetic resonance imaging; (c) no progression to mCRPC prior to CRP; (d) no visceral metastasis; (e) no local treatment for metastatic lesion prior to surgery; and (f) complete clinicopathological data and follow-up information. CRP was performed through open or laparoscopic surgery. Pathological stage was assessed using the American Joint Committee on Cancer 2010 TNM staging system and the Gleason grading system. All patients were treated with ADT until progression to CRPC after CRP. In the event of progression to mCRPC, systemic treatment was delivered by the treating physician using approved drugs for mCRPC. This study was carried out in accordance with the Declaration of Helsinki and approved by the ethical committee of our institution. All patients provided written informed consent.

### Isolation and Classification of CTCs

Isolation and classification of CTCs were performed as described in a previous study using the CanPatrol system (21, 24). Briefly, peripheral blood samples (5 ml, EDTA-anticoagulated) were collected at 12–14 days after surgery, and red blood cell lysis buffer (Sigma–Aldrich) was used to remove erythrocytes within 4 h of collection. The remaining cells were resuspended in PBS with 4% formaldehyde for 5 min, and CTCs were isolated from the remaining cells using a filtration system consisting of a filtration tube containing a membrane (SurExam, Guangzhou, China), a manifold vacuum plate with valve settings (SurExam, Guangzhou, China), an E-Z 96 vacuum manifold (Omega, Norcross, USA), and a vacuum pump (Auto Science, Tianjin, China). An RNA-ISH assay was then performed to identify and classify CTCs based on the target sequences for leukocyte (CD45), epithelial (CK8/18/19 and EpCAM), and mesenchymal (Twist1 and Vimentin) markers; 4´,6-diamidino-2-phenylindole (DAPI) was used to stain nuclei. The RNA-ISH assay was performed in a 24-well plate (Corning), and the cells were analyzed by fluorescence microscopy. CTCs were classified into three subgroups using the CanPatrol CTC enrichment technique: epithelial CTCs (epithelial biomarker positive, CD45 negative), mesenchymal CTCs (mesenchymal biomarker positive, CD45 negative), and biphenotypic epithelial/mesenchymal CTCs (epithelial/mesenchymal marker positive, CD45 negative).

### Statistical Analysis

mCRPC-free survival was defined as the time from initial diagnosis until CRPC. CRPC was defined as castration serum testosterone <50 ng/dl plus either radiological progression using RECIST (Response Evaluation Criteria in Solid Tumors) or three consecutive increases in PSA 1 week apart resulting in two 50% increases over the nadir and a PSA > 2 ng/ml (25). CSS was defined as the time from diagnosis to death from prostate cancer. Frequencies and proportions are used to describe categorical data, and medians and ranges are used to describe continuous data. Correlations of CTC count with continuous and categorical variables were evaluated using Pearson’s test and Kruskal–Wallis H tests, respectively. Univariable Cox regression analyses were performed to assess prognostic factors for mCRPC-free survival and CSS; the significant individual (*p*-value <0.05) or clinically significant prognostic factors were assessed by multivariable Cox regression analysis. Time-dependent receiver operating characteristic (ROC) curve analysis was conducted to evaluate the optimal CTC cutoff point with the maximum Youden index value for predicting mCRPC-free survival and CSS after surgery. The Kaplan–Meier method with the log-rank test was applied to estimate mCRPC-free survival and CSS. SPSS ver. 22.0 (IBM Corporation, Armonk, NY, USA) and the R software environment for statistical computing were used for all statistical analyses. A value of *p* < 0.05 was considered statistically significant.

## Results

### Patient Characteristics

**Figure 1:**
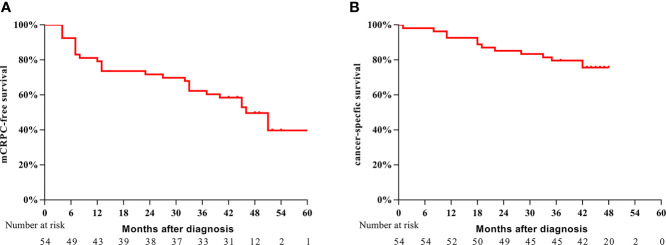
Kaplan–Meier survival analysis of mCRPC-free survival **(A)** and CSS **(B)** for the entire cohort of patients.

Between January 2015 and November 2017, a total of 54 eligible patients with a median age of 68 years (IQR: 61–72 years) were enrolled in this study. The baseline and pathological characteristics of the patients are summarized in **Table 1**. Of the 54 patients, 41 (75.9%) had a pathologic Gleason score ≥8, 17 (31.5%) had pT4 disease, and 24 (44.4%) had lymph node metastasis. The positive surgical margin rate was 66.7%. The median follow-up period was 45 months (IQR: 43–49 months). In the overall cohort, 27 (50.0%) patients experienced progression to mCRPC during the follow-up period; 13 (24.1%) patients died. The 3-year mCRPC-free survival rate was 61.1% (**Figure 1A**), and the 3-year CSS rate was 79.6% (**Figure 1B**). The median time to mCRPC was 46.0 (95% CI: 37.7–54.6) months, though the median time to CSS was not reached. For patients with progression to mCRPC, 17 were treated with abiraterone, and 10 received chemotherapy.

**Table 1:** Patient characteristics.

Variables	Values
Number of patients	54
Age, years	
Median(range)	68 (61-72)
PSA at diagnosis (ng/ml)	
Median(range)	82.2 (38.5-100.4)
Pathologic Gleason score, n (%)	
≤7	13 (24.1)
8	14 (25.9)
≥9	27 (50.0)
Pathologic T stage, n (%)	
T2a-T3a	16 (29.6)
T3b	21 (38.9)
T4	17(31.5)
Pathologic N stage, n (%)	
N0	30 (55.6)
N1	24 (44.4)
Surgical margin, n (%)	
negative	18 (33.3)
positive	36 (66.7)
Number of bone metastases, n (%)	
1-3	36 (66.7)
4-5	18 (33.3)
preoperative ADT therapy, n (%)	
Yes	30 (55.6)
No	24 (44.4)
Postoperative adjuvant ADT, n (%)	54(100)
Postoperative adjuvant RT, n (%)	18(33.3)
First-line therapy for mCRPC (n=27), n (%)	
Abiraterone	17 (63.0)
Chemotherapy	10 (37.0)

PSA, Prostate-specific antigen; ADT, androgen deprivation therapy; mCRPC, metastatic castration-resistant prostate cancer; RT, radiotherapy.

### CTC Detection and Association With Clinicopathological Factors

**Figure 2:**
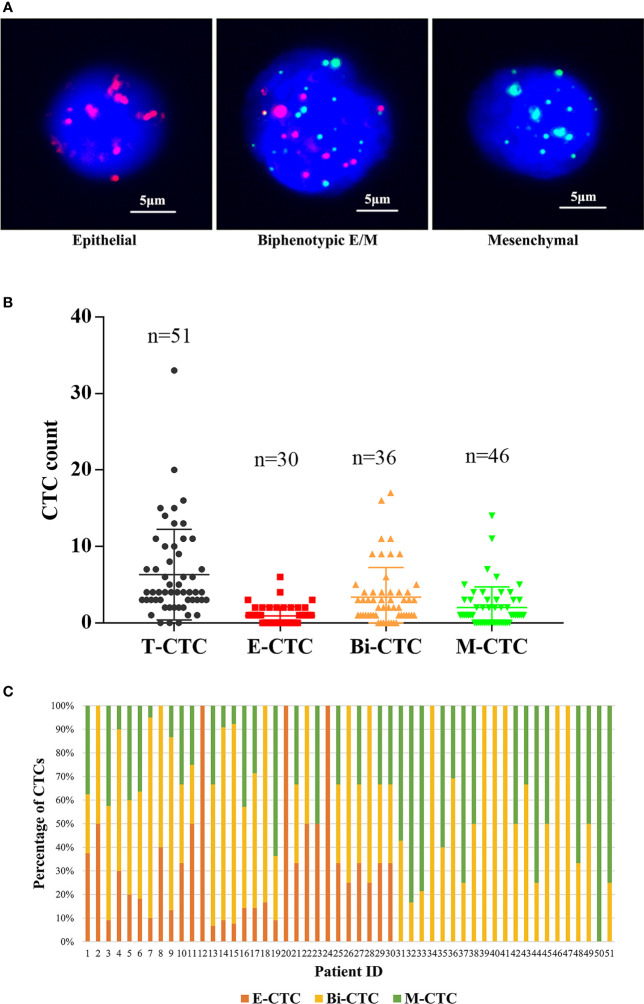
**(A)** Representative fluorescence images of three types of CTCs isolated from the peripheral blood of omHSPC patients based on RNA-ISH staining for leukocytes (CD45, white), epithelial cells (EpCAM and CK8/18/19, red), and mesenchymal cells (vimentin and twist, green). 4´,6-Diamidino-2-phenylindole was used to stain cell nuclei (blue). The scale bar indicates 5 μm. **(B)** Levels of CTC subtypes. **(C)** The distribution of three subtypes of CTCs in each patient.

CTCs were classified into three types through the CanPatrol technique (**Figure 2A**). CTCs were detected in 51 patients (94%), and the median CTC count was 4 (IQR: 3–9). As shown in **Figure 2B**, the detection rates of E-CTCs, M-CTCs, and Bi-CTCs were 56%, 67%, and 85%, respectively. The distribution of the three subtypes in each patient is depicted in **Figure 2C**. After using Pearson’s test and Kruskal–Wallis *H* tests to evaluate the relationship between CTC count and clinical parameters, we found that both T-CTC count and Bi-CTC count correlated positively with lymph node invasion (both *p* < 0.05). In addition, T-CTC and M-CTC counts correlated positively with the number of bone metastases. No significant correlation was found between E-CTC count and clinicopathological factors, and there was no correlation between CTC count and PSA at diagnosis, pathologic Gleason score, or pathologic T stage (**Table 2**).

**Table 2:** Correlation of CTC count and phenotype with clinicopathological variables.

Variable	N	T-CTC	M-CTC	E-CTC	Bi-CTC
		P value	P value	P value	P value
Age					
<70	30	0.30	0.46	0.45	0.43
≥70	24				
PSA at diagnosis	54	0.33	0.46	0.44	0.95
Pathologic Gleason score					
≤7	13	0.12	0.30	0.92	0.34
8	14				
≥9	27				
pT stage					
T2a-T3a	16	0.19	0.066	0.86	0.86
T3b	21				
T4	17				
pN stage					
N0	30	0.014	0.087	0.18	0.027
N1	24				
Surgical margin					
positive	36	0.42	0.38	0.37	0.17
negative	18				
Number of metastases					
1-3	36	0.012	0.009	0.50	0.12
4-5	18				

CTC, circulating tumor cell; T-CTC, total circulating tumor cell; M-CTC, mesenchymal circulating tumor cell; E-CTC, epithelial circulating tumor cell; Bi-CTC, biphenotypic circulating tumor cell; N, number; PSA, Prostate-specific antigen; pT stage, Pathologic T stage; pN stage, Pathologic N stage.

### Univariable and Multivariable Analyses

In univariable analysis, pathologic N stage, number of bone metastases, T-CTC count (continuous and categorical), and M-CTC count (continuous and categorical) were significantly associated with mCRPC-free survival and CSS (**Table 3**). Besides, postoperative adjuvant RT was an independent predictor of mCRPC-free survival (*p* = 0.013, **Table 3**) but not of CSS (*p* = 0.933, **Table 3**). After selecting the significant independent prognostic factors for multivariable Cox regression analysis, T-CTC count (continuous and categorical), M-CTC count (continuous and categorical), and postoperative adjuvant RT were significantly associated with mCRPC-free survival. Additionally, only T-CTC count (continuous and categorical) and M-CTC count (continuous and categorical) were significantly associated with CSS in multivariable Cox regression analysis.

**Table 3:** Univariable and multivariable Cox analysis for mCRPC-free survival and CSS.

Characteristics	mCRPC-free survival				CSS			
	Univariable analysis		Multivariable analysis		Univariable analysis		Multivariable analysis	
	HR (95% CI)	p value	HR (95% CI)	p value	HR (95% CI)	p value	HR (95% CI)	p value
Age	1.030 (0.974-1.091)	0.294	1.087 (0.998-1.184)	0.154^a^	0.995 (0.920-1.076)	0.901	0.983 (0.839-1.152)	0.833^c^
PSA at diagnosis	0.997 (0.993-1.002)	0.256	0.994 (0.987-1.126)	0.256^a^	0.995 (0.986-1.004)	0.270	0.941 (0.902-1.182)	0.125^c^
Pathologic Gleason score								
≤7	Referent	0.045	Referent	0.198^a^	Referent	0.759		
8	1.257 (0.856-2.175)	0.077	3.911 (0.722-7.176)	0.114	3.582 (0.749-7.936)	0.942		
≥9	4.236 (1.445-6.908)	0.014	4.006 (0.858-8.709)	0.078	2.535 (0.815-5.628)	0.940		
Pathologic T stage								
T2a-T3a	Referent	0.233			Referent	0.885		
T3b	2.330 (0.819-6.633)	0.113			2.559 (0.856-7.645)	0.932		
T4	2.341 (0.795-6.893)	0.123			1.745 (0.762-6.379)	0.931		
Pathologic N stage (N0 vs. N1)	3.786 (1.87-10.858)	0.010	0.370 (0.020-6.720)	0.502^a^	0.363 (0.170-0.774)	0.009	3.245 (0.611-17.231)	0.167^c^
Number of metastases (1-3 vs. 4-5)	2.552 (1.164-5.598)	0.019	1.285 (0.482-3.425)	0.617^a^	5.551 (1.705-8.069)	0.004	2.174 (0.548-8.632)	0.270^c^
Positive surgical margin (No vs. Yes)	1.358 (0.586-3.146)	0.475			3.383( 0.741-15.451)	0.116		
Postoperative adjuvant RT (No vs. Yes)	0.390 (0.147-0.835)	0.039	0.113 (0.130-0.422)	0.013^a^	0.950 (0.286-3.156)	0.933		
T-CTC (continuous)	1.123 (1.063-1.187)	<0.001	1.182 (1.052-1.329)	0.035^a^	1.179 (1.082-1.286)	<0.001	1.311 (1.110-1.549)	0.001^c^
E-CTC (continuous)	1.234 (0.931-1.636)	0.144			1.341 (0.906-1.985)	0.143		
M-CTC (continuous)	1.303 (1.152-1.473)	<0.001	1.259 (1.081-1.466)	0.038^a^	1.455 (1.215-1.743)	<0.001	1.386 (1.135-1.693)	0.001^c^
Bi-CTC (continuous)	1.128 (1.036-1.228)	0.005	0.873 (0.730-1.044)	0.137^a^	1.145 (1.026-1.278)	0.015	1.050 (0.913-1.208)	0.491^c^
T-CTC (<5 vs. ≥5)	4.404 (1.946-9.969)	<0.001	4.150 (1.453-7.852)	0.020^b^	5.005 (1.374-8.232)	0.015	3.362 (1.684-8.159)	0.024^d^
M-CTC (<2 vs. ≥2)	3.277 (1.495-7.182)	0.003	3.341 (1.334-8.363)	0.011^b^	3.911 (1.198-6.769)	0.024	3.912 (1.160-7.194)	0.028^d^

mCRPC, metastatic castration-resistant prostate cancer; CSS, cancer specific survival; HR, hazard ratio; CI, confidence interval; CTC, circulating tumor cell; T-CTC, total circulating tumor cell; M-CTC, mesenchymal circulating tumor cell; E-CTC, epithelial circulating tumor cell; Bi-CTC, biphenotypic circulating tumor cell; RT, radiotherapy.

^a^Adjusted for: T-CTC (continuous), M-CTC (continuous), Bi-CTC (continuous), age, PSA at diagnosis, pathologic Gleason score, pathologic N stage, number of metastases and postoperative adjuvant RT.

^b^Adjusted for: T-CTC (<5 vs. ≥5), M-CTC (<2 vs. ≥2), Bi-CTC (continuous), age, PSA at diagnosis, pathologic Gleason score, pathologic N stage, number of metastases and postoperative adjuvant RT.

^c^Adjusted for: T-CTC (continuous), M-CTC (continuous), Bi-CTC (continuous), age, PSA at diagnosis, pathologic N stage and number of metastases.

^d^Adjusted for: T-CTC (<5 vs. ≥5), M-CTC (<2 vs. ≥2), Bi-CTC (continuous), age, PSA at diagnosis, pathologic N stage and number of metastases.

### Prognostic Value of CTC Enumeration and Phenotype

**Figure 3:**
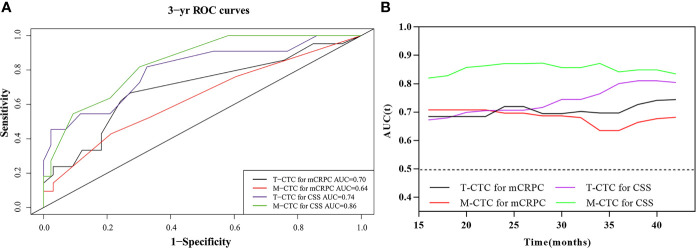
**(A)** Comparison of 3-year predictive efficiency among CTC subtypes according to time‐dependent receiver operating characteristic curve analysis. **(B)** The distribution of the dynamic AUC over time.

Time-dependent ROC analysis was conducted to assess the role of CTCs in prognosis (**Figure 3** and **Table 4**). The results demonstrated that the M-CTC count had higher predictive power than E-CTC and Bi-CTC for mCRPC-free survival (3-year AUC: 0.64, 0.60, and 0.61; **Table 4**) and CSS (3-year AUC: 0.86, 0.58 and 0.67; **Table 4**). Additionally, M-CTC had higher predictive power than T-CTC for CSS (3-year AUC value: T-CTC, 0.74; M-CTC, 0.86; **Figure 3A**), whereas T-CTC had higher predictive power than M-CTC for mCRPC-free survival (3-year AUC value: T-CTC, 0.70; M-CTC, 0.64; **Figure 3A**). **Figure 3B** illustrates dynamic area under the curve (AUC) values over time, from 15 to 40 months, after surgery. According to time-dependent ROC analysis, the maximum Youden index value was applied to calculate the optimal CTC cutoff point, and the results showed that cutoffs of five T-CTCs (≥5 vs. <5) and two M-CTCs (≥2 vs. <2) had a statistically significant impact on mCRPC-free survival and CSS (all *p* < 0.05).

**Table 4:** Comparison of predictive efficiency among different CTC subtypes according to time‐dependent receiver operating characteristic curve analysis.

	AUC for 3 years		AUC for 4 years		AUC for 5 years	
	mCRPC-free survival	CSS	mCRPC-free survival	CSS	m-CRPC-free survival	CSS
E-CTC	0.60	0.58	0.53	0.55	0.61	0.55
Bi-CTC	0.61	0.67	0.60	0.67	0.65	0.67
M-CTC	0.64	0.86	0.76	0.76	0.78	0.76
T-CTC	0.70	0.74	0.74	0.77	0.82	0.77

CTC, circulating tumor cell; T-CTC, total circulating tumor cell; M-CTC, mesenchymal circulating tumor cell; E-CTC, epithelial circulating tumor cell; Bi-CTC, biphenotypic circulating tumor cell.

**Figure 4:**
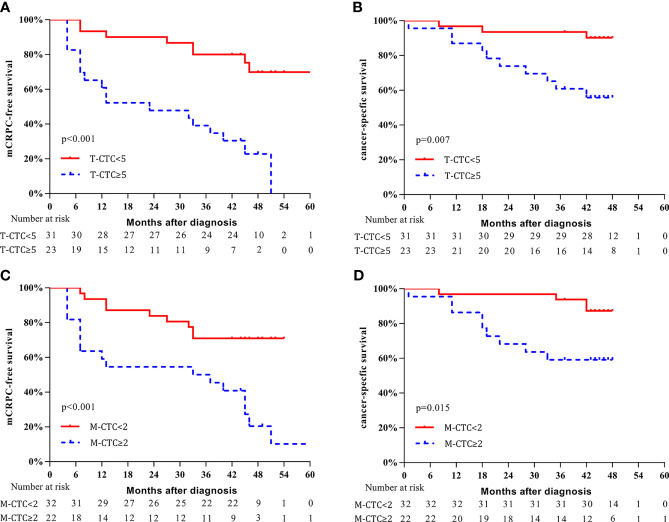
Kaplan–Meier curves of survival outcomes according to CTC phenotype. **(A)** mCRPC-free survival stratified according to T-CTC count. **(B)** CSS stratified according to T-CTC count. **(C)** mCRPC-free survival stratified according to M-CTC count. **(D)** CSS stratified according to M-CTC count.

The CTC cutoff points were then used for survival analysis (**Figure 4**): patients with a T-CTC count ≥5 had significantly shorter 3-year mCRPC-free survival and CSS than those with a T-CTC count <5 (mCRPC-free survival: 39.1% vs. 77.4%, *p* < 0.001, **Figure 4A**; CSS: 60.9% vs. 93.5%, *p* = 0.007, **Figure 4B**). The median time to mCRPC was 23.0 (95% CI: 11.9–46.5) months for patients with a T-CTC count ≥5, whereas the median time was not reached for those with a T-CTC count <5.

Patients with an M-CTC count ≥2 had significantly shorter 3-year mCRPC-free survival and CSS than those with an M-CTC count <2 (mCRPC-free survival: 50.0% vs. 68.8%, *p* < 0.001, **Figure 4C**; CSS: 59.1% vs. 93.8%, *p* = 0.015, **Figure 4D**). The median mCRPC-free survival was 33.0 (95% CI: 16.4-65.2) months for patients with M-CTC count ≥2; for those with M-CTC count <2, the median time was not reached.

**Figure 5:**
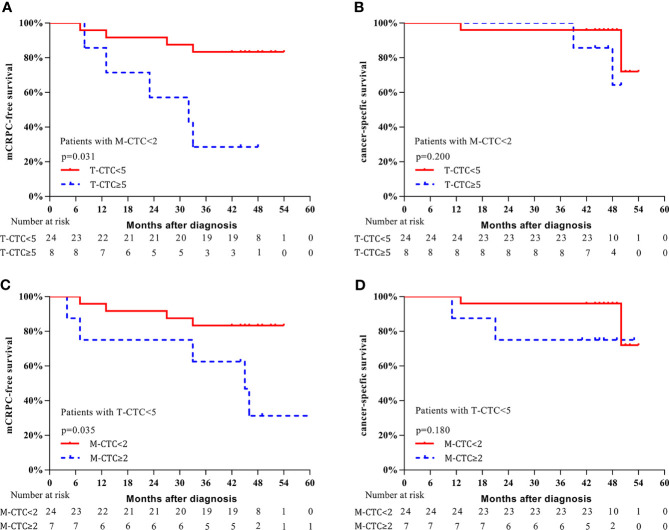
Kaplan–Meier curves of survival outcomes. **(A)** mCRPC-free survival stratified according to T-CTC count in the subgroup of M-CTC<2. **(B)** CSS stratified according to T-CTC count in the subgroup of M-CTC<2. **(C)** mCRPC-free survival stratified according to M-CTC count in the subgroup of T-CTC<5. **(D)** CSS survival stratified according to M-CTC count in the subgroup of T-CTC<5.

Additionally, we conducted subgroup analysis to further investigate the predictive value of CTCs. For the subgroup with M-CTC<2, patients with T-CTC count ≥5 had shorter 3-year mCRPC-free survival (37.5% vs. 79.2%, *p* = 0.031, **Figure 5A**) and a trend of worse CSS (87.5% vs. 95.8%, *p* = 0.200, **Figure 5B**) than those with T-CTC count <5. For the subgroup with T-CTC<5, patients with M-CTC count ≥2 had shorter 3-year mCRPC-free survival (62.5% vs. 80.0%, *p* = 0.035, **Figure 5C**) and a trend of worse CSS (75.0% vs. 96.0%, *p* = 0.180, **Figure 5D**) than those with M-CTC count <2.

## Discussion

Despite increasing acknowledgment of the oligometastatic state in prostate cancer, there is no consensus on its definition. The majority of the published studies regards the prostate cancer with up to 3 to 5 metastatic lesions as the oligometastatic stage (2, 5, 13). In our study, an oligometastatic state was defined as five or fewer bone lesions, with or without suspicious pelvic nodal metastasis. This definition of oligometastatic prostate cancer is consistent with previous studies that investigated the impact of radical prostatectomy (3, 5). A phase 3 trial found that prostate-directed radiation can improve survival outcomes in patients with a low metastatic burden (26). Despite no hard evidence for the survival benefit of CRP in oligometastatic prostate cancer, several retrospective and prospective studies have suggested that CRP reduces the risk of clinical progression and improves long-term survival in patients with oligometastatic prostate cancer (3–6, 13). Currently, PSA kinetics are commonly used to follow disease progression, but they cannot be utilized to predict the prognosis of omHSPC patients well because of inherent limitations (24). Thus, it is extremely important to identify an independent prognostic factor to help in therapeutic decision-making for omHSPC patients who undergo CRP.

It is reported that the number of metastatic lesions (≥10 vs. 10) is not an independent predictor of mCRPC-free survival for metastatic hormone-sensitive prostate cancer (mHSPC) patients (24). One recent study also found that the number of metastases was not associated with overall survival in oligometastatic patients with prostate cancer who were treated with local and metastatic curative radiotherapy (27). In the present study, we found that the number of bone metastases was not a significant predictor of mCRPC-free survival or CSS in omHSPC patients. These findings suggest that the number of metastases might not be the primary prognostic factor for prostate cancer and support investigation of an independent biomarker for omHSPC when the metastatic burden varies from one to five. Since the discovery of CTCs, enormous attention has been given to investigating their potential as prognostic and treatment response biomarkers for mCRPC patients (11, 28). In this study, we detected the level of CTCs, characterized their phenotype, and further explored the clinical significance of CTCs in omHSPC patients who underwent CRP.

Although the CellSearch system has been widely used to detect CTCs depending on tumor cell epithelial markers such as EpCAM and CK, it fails to detect CTCs undergoing EMT. Thus, we used the CanPatrol enrichment technique, a filter-based method that uses a combination of epithelial and mesenchymal markers, to isolate and identify different phenotypes of CTCs; Bi-CTCs and M-CTCs can be simultaneously detected based on this novel system in addition to E-CTCs. The detection rate of CTCs was 94%, higher than that using the CellSearch system for mHSPC patients (38%–48.5%) (13, 29). Several reports have indicated an increase in CTC count with progression of the disease stage; for instance, the detection rate of CTCs was 0%–10% for healthy volunteers (30, 31), 5%–38.4% for nonmetastatic high-risk prostate cancer patients (30, 31), and 80% for mCRPC patients (15). In our cohort, all patients had metastatic prostate cancer, with 75.9% having disease with a Gleason score ≥8, and the aggressive clinicopathological characteristics might be another reason for the higher detection rate of CTCs.

CTCs have been proposed as prognostic biomarkers to predict treatment response and survival outcomes in mCRPC patients. A phase III trial (SWOG S0421) recruiting mCRPC patients treated with docetaxel indicated that baseline CTC count ≥5 was associated with shorter OS (28). Scher et al. (11) also found that a biomarker panel containing the CTC number and LDH level could be used as a surrogate for survival in mCRPC patients treated with abiraterone. The clinical significance of CTCs in mHSPC patients has recently been documented (13, 24). A prospective study of omHSPC patients receiving CRP demonstrated that CTC count ≥2 is associated with shorter mCRPC-free survival and OS (13). Similarly, a phase III prospective randomized trial (SWOG S1216) of ADT combined with orteronel or bicalutamide for mHSPC patients found that the baseline CTC count was highly prognostic of 7-month PSA and 2-year PFS (32). Consistent with the literature, our study found the T-CTC count to be an independent predictor of mCRPC-free survival and CSS in multivariable Cox regression analysis; in addition, an optimal cutoff of 5 had a statistically significant impact on survival outcomes. Overall, T-CTC count ≥5 was associated with early progression to mCRPC and shorter CSS, in accordance with the literature (11, 29).

It is well known that CTCs adopt a mesenchymal phenotype in the process of EMT, which endows cells with multiple malignant traits (16, 17). Recently, several studies have reported the significance of M-CTCs in a variety of malignancies, including breast (19), liver (21), colorectal (22), and prostate (24) cancer. These studies demonstrate that M-CTCs are significantly associated with early recurrence, progression, and metastasis (19, 22, 24). By using the CanPatrol system to detect M-CTCs, the current study found that a higher M-CTC count was associated with a higher number of metastases, though no significant correlation between E-CTCs and the number of metastases was detected, which might be attributed to the higher invasion and migration potential of M-CTCs than E-CTCs (16, 17). According to time-dependent ROC analysis, we found that the M-CTC count had higher predictive power than E-CTC and Bi-CTC counts for mCRPC-free survival and CSS. Indeed, E-CTCs and Bi-CTCs did not show a significant relationship with prognosis for omHSPC patients, whereas M-CTCs were independently associated with mCRPC-free survival and CSS. Specifically, patients with M-CTC count ≥2 had worse mCRPC-free survival and CSS. We also explored the predictive value of M-CTC in the subgroup of patients with T-CTC count<5, and the results again demonstrated that those with M-CTC count ≥2 had a significantly shorter time to mCRPC and a trend of worse CSS. The reason that there was no statistical significance for CSS may be due to the small sample size. Thus, a prospective study with a large sample size is needed for further exploration. Our findings demonstrate the prognostic significance of M-CTCs for omHSPC, even in patients with T-CTC count <5, supporting the potential use of M-CTCs as a novel biomarker for omHSPC patients who undergo CRP.

It is reported that the incidence rate of positive surgical margins (PSM+) ranges from 72.7% to 78.9% in oligometastatic prostate cancer patients treated with CRP (2, 3, 5, 13). In our study, the rate of PSM+ was 66.7%, which was in accordance with the data in these previous studies. In addition, 50% of the patients with PSM+ in our study had received postoperative adjuvant RT plus ADT, whereas the others received ADT alone. Overall, postoperative adjuvant RT is an independent predictor of mCRPC-free survival, though there was no significant association between RT and CSS. The results need to be validated in larger prospective studies.

There were some limitations in our research. First, this was a retrospective study, and the results need to be validated in future prospective studies. Second, the small cohort size might have caused bias and influenced the results of multivariable analyses. Third, the impact of additional therapy (chemotherapy and abiraterone) after mCRPC could not be adjusted in multivariable analysis because the number of mCRPC patients was small and the patients were treated differently according to their physician’s choice. Fourth, the optimal time interval for CTC quantification after surgery has not been conclusively established. Dynamic monitoring of CTC changes might be essential in the future (13).

## Conclusion

This study is the first to demonstrate that both T-CTC count ≥5 and M-CTC count ≥2 are independent predictors of early progression to mCRPC and shorter CSS after CRP for omHSPC patients. The findings support the use of CTC quantification and phenotype characterization as a prognostic biomarker to identify patients’ cases progressing early and to select intensive treatment after surgery. The results need to be validated in prospective, randomized trials in the future.

## Data Availability Statement

The original contributions presented in the study are included in the article/supplementary material. Further inquiries can be directed to the corresponding authors.

## Ethics Statement

The studies involving human participants were reviewed and approved by the Ethics Committee at the Tenth People’s Hospital of Shanghai (SHSY-IEC-4.1/20-22/01). The patients/participants provided their written informed consent to participate in this study.

## Author Contributions

Conception and design: XY and BY. Acquisition of data: GY, JX, and WG. Analysis and interpretation of data: GY, SZ, JY, RW, and CG. Writing, review, and/or revision of the manuscript: GY, JX, SZ, and BY. Administrative, technical, or material support: XY and BP. Study supervision: LY, BP, XY, and BY. All authors contributed to the article and approved the submitted version.

## Funding

This work was partly supported by the National Natural Science Foundation of China (Grant No. 31570993) and Shanghai Pujiang Program (Grant No. 15PJ1407000).

## Conflict of Interest

The authors declare that the research was conducted in the absence of any commercial or financial relationships that could be construed as a potential conflict of interest.

## Publisher’s Note

All claims expressed in this article are solely those of the authors and do not necessarily represent those of their affiliated organizations, or those of the publisher, the editors and the reviewers. Any product that may be evaluated in this article, or claim that may be made by its manufacturer, is not guaranteed or endorsed by the publisher.
